# Supplementary material for: The incidence of chronic pain following Cesarean section and associated risk factors: A cohort of women followed up for three months
Source: PLoS One. 2020 Sep 4;15(9):e0238634. doi: 10.1371/journal.pone.0238634 (PMC7473578; doi:10.1371/journal.pone.0238634)
Supplement: S1 Table — (PDF) [file pone.0238634.s001.pdf]

S1 Table: Medication received postoperatively during hospitalization.

**Medication received as pain relief**[a]	**Women (n=620)**	
	**n**	**%**
**Simple analgesics**[b]	612	99.7
Dipyrone	607	99.2
Acetaminophen	5	0.8
**NSAIDs**[c]	571	93.3
Diclofenac sodium	568	99.5
Tenoxicam	111	19.4
**Opioids**[b]	5	0.8
Tramadol	5	100.0
**Combination drugs**[b]	5	0.8
Caffeine + carisoprodol + diclofenac sodium + acetaminophen	4	80.0
Codeine phosphate + acetaminophen	1	20.0

[a] The women may have taken more than one type of pain relief medication; [b] Data missing=6; [c] Data missing=8; NSAIDs: Non-steroidal anti-inflammatory drugs.
