# Supplementary material for: The incidence of chronic pain following Cesarean section and associated risk factors: A cohort of women followed up for three months
Source: PLoS One. 2020 Sep 4;15(9):e0238634. doi: 10.1371/journal.pone.0238634 (PMC7473578; doi:10.1371/journal.pone.0238634)
Supplement: S2 Table — (PDF) [file pone.0238634.s002.pdf]

S2 Table: Use of medication by the women with pain on the 7th day following surgery (n=434).

**Pain relief medications**[a]	**Women (n=434)**[b]	
	**n**	**%**
**Use of pain relief medication**	409	95.8
**Simple Analgesics**	319	78.0
Dipyrone	306	95.9
Acetaminophen	16	5.0
**NSAIDs**	335	81.7
Diclofenac sodium	328	97.9
Nimesulide	6	1.8
Naproxen	1	0.3
Ibuprofen	1	0.3
**Combination drugs**	8	2.0
Caffeine + carisoprodol + diclofenac sodium + acetaminophen	2	25.0
Dipyrone + promethazine hydrochloride + adiphenine hydrochloride	5	62.5
Carisoprodol + diclofenac sodium + acetaminophen + caffeine	1	12.5

[a] The women may have taken more than one type of pain relief medication; [b] Data missing=7; NSAIDs: Non-steroidal anti-inflammatory drugs.
