# Supplementary material for: The incidence of chronic pain following Cesarean section and associated risk factors: A cohort of women followed up for three months
Source: PLoS One. 2020 Sep 4;15(9):e0238634. doi: 10.1371/journal.pone.0238634 (PMC7473578; doi:10.1371/journal.pone.0238634)
Supplement: S3 Table — (PDF) [file pone.0238634.s003.pdf]

S3 Table: Use of medication by the women with pain on the 30th day following surgery (n=173).

Pain relief medications[a]	Women (n=173)[b]	
	n	%
**Use of pain relief medication**	58	36.0
**Simple Analgesics**	39	67.2
Dipyrone	34	87.2
Acetaminophen	5	12.8
**NSAIDs**	26	44.8
Diclofenac sodium	24	92.3
Ibuprofen	1	3.8
Nimesulide	1	3.8
**Combination drugs**	1	1.7
Dipyrone + orphenadrine citrate + anhydrous caffeine	1	100.0

[a] The women may have taken more than one type of pain relief medication; [b] Data missing=12; NSAIDs: Non-steroidal anti-inflammatory drugs.
