# Supplementary material for: The incidence of chronic pain following Cesarean section and associated risk factors: A cohort of women followed up for three months
Source: PLoS One. 2020 Sep 4;15(9):e0238634. doi: 10.1371/journal.pone.0238634 (PMC7473578; doi:10.1371/journal.pone.0238634)
Supplement: S4 Table — (PDF) [file pone.0238634.s004.pdf]

S4 Table: Use of medication by the women with pain on the 60th day following surgery (n=147).

**Pain relief medications**[a]	**Women (n=147)**[b]	
	**n**	**%**
**Use of pain relief medication**	22	15.5
**Simple Analgesics**	12	54.5
Dipyrone	11	91.7
Acetaminophen	1	8.3
**NSAIDs**	10	45.5
Diclofenac sodium	9	90.0
Nimesulide	1	10.0
**Combination drugs**	1	4.5
Caffeine + carisoprodol + diclofenac sodium + acetaminophen	1	100.0

[a] The women may have taken more than one type of pain relief medication; [b] Data missing=5; NSAIDs: Non-steroidal anti-inflammatory drugs.
