# Supplementary material for: The incidence of chronic pain following Cesarean section and associated risk factors: A cohort of women followed up for three months
Source: PLoS One. 2020 Sep 4;15(9):e0238634. doi: 10.1371/journal.pone.0238634 (PMC7473578; doi:10.1371/journal.pone.0238634)
Supplement: S5 Table — (PDF) [file pone.0238634.s005.pdf]

S5 Table: Use of medication by the women with pain on the 90th day following surgery (n=118).

**Pain relief medications**[a]	**Women (n=118)**[b]	
	**n**	**%**
**Use of pain relief medication**	19	17.0
**Simple Analgesics**	11	57.9
Dipyrone	10	90.9
Acetaminophen	1	9.1
**NSAIDs**	7	36.8
Diclofenac sodium	5	71.4
Nimesulide	1	14.3
Ibuprofen	1	14.3
**Combination drugs**	2	10.5
Caffeine + carisoprodol + diclofenac sodium + acetaminophen	1	50.0
Dipyrone + adiphenine hydrochloride + promethazine hydrochloride	1	50.0

[a] The women may have taken more than one type of pain relief medication; [b] Data missing=6; NSAIDs: Non-steroidal anti-inflammatory drugs.
